# A proposed checklist for climate-friendly sport and exercise programmes

**DOI:** 10.1093/eurpub/ckac073

**Published:** 2022-08-26

**Authors:** Karim Abu-Omar, Sven Messing, Antonina Tcymbal, Tobias Fleuren, Diana Richardson, Stephen Whiting, Peter Gelius, Kremlin Wickramasinghe

**Affiliations:** Department of Sport Science and Sport, Friedrich-Alexander-Universität Erlangen-Nürnberg, Erlangen, Germany; Department of Sport Science and Sport, Friedrich-Alexander-Universität Erlangen-Nürnberg, Erlangen, Germany; Department of Sport Science and Sport, Friedrich-Alexander-Universität Erlangen-Nürnberg, Erlangen, Germany; Department of Sport Science and Sport, Friedrich-Alexander-Universität Erlangen-Nürnberg, Erlangen, Germany; Department of Sport Science and Sport, Friedrich-Alexander-Universität Erlangen-Nürnberg, Erlangen, Germany; WHO European Office for the Prevention and Control of Noncommunicable Diseases, Moscow, Russian Federation; EPIUnit—Instituto de Saúde Pública, Universidade do Porto, Porto, Portugal; Department of Sport Science and Sport, Friedrich-Alexander-Universität Erlangen-Nürnberg, Erlangen, Germany; WHO European Office for the Prevention and Control of Noncommunicable Diseases, Moscow, Russian Federation

## Abstract

**Background:**

Fighting the climate crisis is the greatest challenge of our time and will touch all aspects of people’s lives. In this context, the United Nations (UN) have called on the sport sector to reduce its negative impacts on the environment and show ‘climate leadership’. While some efforts have already been made with regards to mega sport events, there is still a dearth of approaches on limiting the climate impact of recreational sport and exercise programmes.

**Methods:**

Based on the UN-Framework ‘Sports for Climate Action’, literature reviews and additional desk research, a checklist to support local level stakeholders in providing climate-friendly sport and exercise programmes was developed.

**Results:**

The proposed checklist consists of five dimensions that need to be considered when designing and offering a climate-friendly sport and exercise programme: (i) active transport to exercise programmes, (ii) the carbon footprint of different types of exercises, (iii) low carbon sport clothing and equipment, (iv) instructors as champions for climate action and (v) advertising and communication. These five dimensions result in a 16-item checklist that supports the planning, advertising, implementation and evaluation of climate-friendly sport and exercise programmes.

**Conclusions:**

The proposed checklist intends to facilitate the development of climate-friendly sport and exercise programmes. However, additional work is needed to test the implementation of the checklist at the local level. While the sport sector can make its own contributions to reduce its climate impact, intersectoral action is needed to improve infrastructure for active transport and to build sustainable sport facilities.

## Introduction

The Intergovernmental Panel on Climate Change reported that the next years will determine if humanity can limit global warming to at around 1.5°C.^1^ At the same time, we live in what has been described as the sixth age of mass extinction,^2^ and our natural environment is rapidly deteriorating.^3^ Scientists and spiritual leaders such as Pope Francis and the Dalai Lama described the dramatic consequences of climate change from different perspectives and highlighted that there is an urgent need for climate action.^4–6^ From a public health perspective, empirical data indicate that there is a causal relationship between anthropogenic climate change and heat-related mortality,^7^ and a recent modelling study showed that increasing efforts to meet the 1.5 degree goal have the potential to protect future human health.^8^

In light of these and other global challenges, the United Nations (UN) adopted the 2030 Agenda for Sustainable Development that includes a call to take ‘urgent action on climate change’ and 17 Sustainable Development Goals (SDGs).^9^ With respect to the detrimental effects of a sedentary lifestyle on health, sport and exercise programmes are instrumental in achieving SDG 3 ‘Good Health and Well-Being’.^10^ Until now, however, there has been limited discussion in the public health community on how sport and exercise programmes can contribute to the other SDGs. As pointed out recently by Nigg and Nigg, physical activity is also connected to the goals on climate action (SDG 13), sustainable cities and communities (SDG 11) and responsible consumption and production (SDG 12).^11^ The links between physical activity and the SDGs are also highlighted in an appendix to the World Health Organization’s Global Action Plan on Physical Activity (GAPPA).^12^ While the SDGs focus on ecological, social and economic sustainability equality, to this paper, ecological sustainability is paramount.

Parts of the sports sector have recognized the importance of climate action and first initiatives exist that aim to reduce the impact of sports on the climate. Prominently, the UN Sports for Climate Action Initiative acknowledges the sport sectors’ contribution to climate change and highlights the opportunity to become a climate leader. The organization’s Sport for Climate Action Framework aims to combat climate change and to use sport as a tool to drive climate awareness and action. Therefore, it calls on all stakeholders in sport to apply the following principles in their daily practice^13^:

1. Undertake systematic efforts to promote greater environmental responsibility
2. Reduce overall climate impact
3. Educate for climate action
4. Promote sustainable and responsible consumption
5. Advocate for climate action through communication

While these principles are already being implemented on the elite sports level,^14^ more guidance is urgently needed on how to apply them on a local or grassroots level. The local level has been called pivotal for efforts to reduce greenhouse gas emissions^15^ and it also has enormous public health potential for the promotion of physical activity through the provision of sport exercise programmes.^12^

The objective of this paper is to present the development of a brief and easy to implement checklist for climate-friendly sport exercise programmes. This checklist aims to support programme planners and decision makers at the local level that plan to provide climate-friendly sport and exercise programmes. The term ‘climate friendly sport and exercise programs’ refers to programmes that apply the five principles of the Sport for Climate Action Framework in their daily practice. This paper focuses on sport and exercise programmes at the local level that are commonly run by communities, sport clubs, churches, or other welfare, non-profit or for-profit organizations and addresses programme planners and decision makers within these organizations.

## Methods

Methodologically, the development of the checklist strives to be within the paradigm of transformation (or sometimes called transformative) research.^16^ Within this paradigm, important quality criteria related to the research process are transparency and reflexivity. In regard to transparency, an unsystematic review of the literature and expert rating were conducted in order to develop the proposed checklist. Thus, to a certain degree, the proposed checklist is normative, in the sense of interconnecting local level sport and exercise programmes to climate change. Being reflexive, we acknowledge that the development of the proposed checklist is driven by the perceived urgency to act on the matter of climate change, which requires that also local level sport and exercise programmes reduce their carbon footprint. As all transformative research, the proposed checklist is intended to be trustworthy, and to produce as outcomes social and scientific impact.^16^

The checklist was developed in a three-step process. First, dimensions of environmental sustainability that intersect with recreational sport and exercise programmes were defined using the UN Sports for Climate Action Framework.^13^ This framework invites sport organizations to sign up to five action areas to combat climate change and to demonstrate ongoing process over time. The checklist can be used as a tool to support systematic efforts of sport organizations to promote greater environmental responsibility, according to the first principle of the framework, but also of other stakeholders. All dimensions of the checklist are linked to the five principles of the Sport for Climate Action Framework (table 1).

**Table 1:** The checklist as a tool to implement the UN Sports for Climate Action Framework

UN Sports for Climate Action Framework	Dimensions of the checklist
Principle 1: Undertake systematic efforts to promote greater environmental responsibility	*[Cross-cutting principle, checklist can be part of systematic efforts to promote greater environmental responsibility]*
Principle 2: Reduce overall climate impact	Dimension 1: (Active) transport to exercise programmes and carbon emissions
	Dimension 2: The carbon footprint of different types of exercises
Principle 3: Educate for climate action	Dimension 4: Instructors as champions for climate action
Principle 4: Promote sustainable and responsible consumption	Dimension 3: (Low carbon) sport clothing and equipment
Principle 5: Advocate for climate action through communication	Dimension 5: Advertising and communication

In a second step, the relevance of each dimension for sport and exercise programmes was summarized based on the scientific evidence. In order to do so, we drew on a number of relevant current literature reviews that investigated the interconnections between physical activity, sport, and climate,^17^^,^^18^ and conducted additional desk research.

The third step was the development of an easy-to-implement 16-item checklist that is based on the previously defined dimensions as well as on scientific evidence. Based on this checklist, programme planners and decision makers can align their efforts in implementing WHO’s GAPPA^12^ with a contribution to achieving UN’s SDGs^9^ and implementing the UN Sport for Climate Action Framework.^13^

## Results

In this section, we describe the results of step two (scientific evidence of each dimension) and three (proposed checklist) of the methodology employed.

### (Active) transport to exercise programmes and carbon emissions

Interconnections between greenhouse gas emissions and active transport were investigated in various studies, showing that active transport is associated with a reduction in CO_2_ emissions.^18^ According to the UN Economic Commission for Europe, in many countries, transport is responsible for more than 30% of the energy consumed by end users such as households, industry and agriculture.^15^ In the European Union, the transportation sector contributes to almost a quarter of greenhouse gas emissions, and strategies have already been put into place to drastically reduce these emissions by mid-century.^19^ The detrimental effects of air pollution from fossil fuel on health combustion are well documented, and contribute to millions of deaths per year globally.^20^^,^^21^

Shifting towards active transport (walking, cycling) helps to reduce air pollution, which has additional health benefits for the local population.^11^^,^^22^ From an economic perspective, cost–benefit analyses reveal another advantage of active transport: While driving a car is related to external costs of 0.11 €/km (e.g. due to costs for infrastructure construction and maintenance), cycling and walking lead to financial benefits of 0.18 €/km and 0.37 €/km, respectively (e.g. due to health benefits that result in savings to the healthcare system).^23^ Based on WHO’s Health Economic Assessment Tool, studies have shown in a different context (active commuting to work) that commuting by cycling and walking on a regular basis results in an enormous health economic benefit at the population level, e.g. 780 million Euro per year in Scotland.^24^ Furthermore, the Pan-European Master Plan for Cycling Promotion highlights that cycling is the fastest and most efficient mode of transport for distances of up to 5 km, and very space-efficient.^25^ A shift towards active transport is heavily influenced by the local environment, and cities such as Copenhagen have demonstrated that the share of walking and cycling can surpass the share of car use.^26^ Additionally, active transportation to and from exercise programmes can help people to achieve WHO physical activity guidelines, which can result in important individual health benefits.^27^^,^^28^ In addition to walking or cycling, several studies have shown that even using public transport can promote physical activity, as walking is required to access and egress the public transport network and to transfer between routes or modes.^29^^,^^30^

In order to support active travel, it is important for programme planners to select locations for their exercise programmes carefully. This has three aspects. First, an exercise programme should be conducted at a place that is easy to reach by active or public transport. Preferably, it is at a location with nearby public transport stations and can also be reached safely by walking and biking. Second, programme planners should reflect on the target group for the exercise programme and whether this population lives in walking and biking proximity of the site of the exercise programme. Third, planners need to balance the need for more specialized exercise programmes, designed for specific target groups (e.g. exercise programmes for individuals with rheumatoid arthritis), with the potentially longer commuting distances of participants they might result in. A programme that is inclusive and allows diverse participants (e.g. wheel chair basketball for everybody) might result in shorter average commutes.

### The carbon footprint of different types of exercises

Studies have investigated the carbon footprint of different types of sport and found out that their carbon footprint varies.^18^ Emissions were especially caused by the transportation required for sport practices, but also by the energy consumption (heating, cooling) of sport and exercise facilities.^18^^,^^31^ Wicker calculated that individual sports, such as gym-based exercise programmes, carry a rather small carbon foot print (228 kg per person per year) when compared with the average carbon footprint of 20 different sports (844 kg).^31^ Popular sports such as water aerobics, on the other hand, have a rather large carbon footprint, due to the high energy consumption of heated indoor pools.^31^ Boussabaine and colleagues estimated that heated indoor swimming pools consume 1.250–1.750 kWh/m^2^ of energy per year, compared with the substantially lower energy consumption of gyms (210–350 kWh/m^2^ per year).^32^

In order to minimize the carbon footprint of exercise programmes, programme planners should be aware of the carbon footprint of different activities and offer preferably types of sports with a low carbon footprint. Additionally, multipurpose facilities can help to reduce the carbon footprint of an exercise programme, especially when they are used by as many people as possible. In some regions of the world with a well-tempered climate, it might also be possible to offer outdoor exercise programmes, which do not require the cooling or heating of facilities (e.g. replacing a water-aerobics programme in a heated indoor pool with an outdoor exercise class). In this regard, classes could be offered based on seasonality (e.g. outdoor Yoga in summer, Nordic Walking in winter).^17^

Making the switch to outdoor exercise programmes could also increase the health benefits for participants. Recent reviews have documented the additional health benefits of outdoor sports and, to a limited extent, of outdoor gyms.^33^^,^^34^

### (Low carbon) sport clothing and equipment

There are also interconnections between climate change and the sports products industry, which has grown significantly over the years. The climate impacts of sports products can be understood as carbon emissions from the production and transportation of goods and materials, and from their operational use and disposal.^14^ The industry is constantly evolving due to the introduction of new technologies and designs, as well as the need to meet the diverse demands and changing personal preferences of customers.^35^ As a result, product life cycles are shortened with highly detrimental social and environmental impacts.^36^

In this regard, it is important to raise awareness among participants in exercise programmes about ways to reduce the negative environmental impact of the consumption of sporting goods on the surrounding environment. Due to its detrimental effect on the environment,^37^ if possible, participants should be instructed that new or fancy exercise clothes are not necessary to take part in classes. They may also be advised to buy used items, give preference to local manufactures, pay attention to labels and give priority to items made from recycled (‘secondary materials’) or eco-friendly materials, if possible. To extend the life of sporting goods, participants could be offered to rent and/or to share equipment and goods from sport clubs. They should also be informed about where used clothes and equipment can be donated or recycled. These concepts of a sharing economy have demonstrated to have positive environmental effects by limiting resources and the carbon footprint of products.^38^ Obviously, sharing and reducing equipment has to be balanced with the need for appropriate clothing and safety requirements for certain sports.

### Instructors as champions for climate action

Social influence plays an important role in the sustainable changes in consumer behaviour.^39^ The presence and behaviours of others influence the individual. This influence occurs through social learning, a process when new behaviours can be acquired by observing and imitating others.^40^ Social learning occurs more quickly when there is an authoritative person who acts as a role model. In the case of exercise programmes, such a person might be a trainer.^41^^,^^42^

It is easier for participants to adopt and maintain climate-friendly habits with the support and guidance of an instructor who exemplifies environmentally oriented behaviour and is knowledgeable about this topic. Exercise programme instructors should exemplify all the principles they advise participants to adhere to.^41^

Such principles should also include eating a healthy diet, since it is a key factor in maintaining good health and at the same time, nutrition has a crucial impact on environmental protection.^43^ The EAT-Lancet Commission named food as the ‘single strongest lever to optimize human health and environmental sustainability on Earth’.^44^ Furthermore, plastic pollution has an impact on environmental protection. In particular, the use of single-plastic use products, such as disposable plastic bottles have a negative impact on environmental pollution.^45^

Organizations should therefore encourage and support instructors, if possible, to use active modes of transport to commute to programme sites and be knowledgeable of the carbon footprint of different types of exercise. The instructors’ clothing and equipment should be made of eco-friendly and recyclable materials. Instructors should be made aware that their eating habits and the use of reusable water bottles will influence the behaviour of the participants in their programmes. Interconnections between exercise and sustainability should be integrated in the education of exercise instructors.

### Advertising and communication

One of the main objectives of the UN Sports for Climate Action Initiative is for stakeholders in sports to use their power and influence to inspire positive climate action.^13^ Exercise programme planners can achieve this in part through sustainable advertising and communication. This encompasses both advertising and promoting sustainable practices (e.g. using active transport to commute to exercise programmes) and ensuring that the advertisement methods themselves are as environmentally friendly as possible. Active transport to exercise programmes can be encouraged by limiting the amount of parking spots for cars and/or providing adequate and secure bike parking.^46^ Information about the available nearby parking should be given to participants ahead of time to help reduce additional driving time looking for a parking spot. Programme planners should also inform participants on how to reach the programme site with public transportation (e.g. providing the nearest bus stop to the programme site). Information about connecting biking and walking paths can also be helpful for individuals who plan to commute with active transport. Similarly, participants can be informed about the carbon footprint of different types of exercise, low carbon sport clothing and equipment, and a healthy diet. Obviously, such measures might be more feasible in urban and densely populated areas. In rural areas where no or limited public transport is available, the carbon footprint of exercise programmes have to be balanced with the health and social effects they have.

Programme planners should also consider the environmental impact of the forms of advertisement they use to promote their programmes. Carbon emissions of printed advertisements (e.g. newspapers, magazines and flyers) are associated with the production and distribution of paper materials, the bulk of which are exported.^47^ When defining the carbon footprint of an object, a CO_2_ equivalent is generally used to convert the total climate change impact of all the greenhouse gases emitted by an object to the equivalent amount of carbon dioxide with the same potential impact. Using this measurement, Berners-Lee calculated that the carbon footprint associated with a yearlong subscription to a daily newspaper is equivalent to that of a one-way flight from London to Madrid (270 kg CO_2_ equivalents).^48^ In comparison, a standard sized email sent out daily for a year would have a carbon footprint of around 1.44 kg CO_2_ equivalents.^48^ Advertising exercise programmes through digital means (e.g. emails, social media ads, mobile apps) can help reduce greenhouse gas emissions by avoiding the over-production and waste associated with commercial print.^49^ While presumably being more sustainable, digital advertising may not be appropriate for all target groups (e.g. elderly individuals). If printed forms of advertisement are desired, efforts should be made to use recycled, eco-friendly paper.

### Checklist for climate-friendly exercise programmes

These five dimensions need to be considered in all phases of programme planning, implementation and evaluation of a climate-friendly sport and exercise programme. The easy-to-understand checklist can be used as a tool for the self-monitoring of organizations that offer sport and exercise programmes (table 2).

**Table 2:** Checklist for climate-friendly exercise programmes

No.	Question	Yes	Rather yes	Rather no	No
Planning					
1	Does the exercise programme take place outdoors, if seasonality permits?				
2	If the exercise programme takes place indoors, is heating or air-conditioning only used when necessary?				
3	Does the type of exercise/sports that was chosen require equipment that is durable?				
4	Is the site of the exercise programme located nearby public transport stations and can it easily be reached by public transport at the time the programme is offered?				
5	Can the site of the exercise programme be reached safely and easily by walking and biking, including bike storage?				
6	Does the programme target people who live in walking and biking proximity to the site of the exercise programme?				
Advertising the programme					
7	Are people informed about and encouraged to use environmentally friendly transport options to commute to the programme site?				
8	Are people advised on a casual dress code, options for buying recycled, eco-friendly or used items, and do organizers provide options for renting or sharing sports equipment?				
9	Is the carbon footprint of advertising the exercise programme minimized?				
Implementation of the programme					
10	Is the instructor a champion for health and climate action regarding active transport, low carbon sport clothing and equipment, healthy snacks and reusable water bottles?				
11	Is the instructor briefed on how people can get to the programme site by public transport, cycling or walking and the health benefits of active transport?				
12	Is the instructor briefed on the carbon footprint of different types of exercises?				
Evaluating and improving the programme					
13	Is information gathered on the participant’s addresses, to assess whether there would be a more suitable location for the exercise programme?				
14	Is information gathered on how people commute to the programme in order to assess whether the number of people using active modes of transport is increasing?				
15	Is the demand for a garage sale/drop off for second-hand clothing or sport equipment assessed?				
16	Were participants asked for suggestions on how to make the programme more climate friendly?				

## Discussion

We have attempted to describe how sport and exercise programmes can be made more climate friendly. Based on the UN Sports for Climate Action Framework,^13^ we identified active transport to sport and exercise programmes, the carbon footprint of different types of exercises, sport clothing and equipment, instructors as champions for climate action, and advertising and communication as important levers to consider as potential drivers to reduce the ecological footprint of sport and exercise programmes. Our analysis has shown that there are ways to make exercise programmes more climate friendly and by doing so, these changes increase rather than limit the potential (health) benefits of exercise programmes (e.g. outdoor activities, active transport, less focus on what people wear to promote equality). This is an important and highly relevant message. However, implementing these changes raises a number of issues, also regarding the relationship between sport and climate action.

### Implementation of the checklist

The checklist is designed to be utilized by programme planners and decision makers on the local level. However, until now, we have not addressed by whom and how often it should be completed. Doing so would also require additional information to be given to programme planners in order to answer the questions of the checklist (e.g. regarding the type of sport equipment that is durable/sustainable). Also, the question remains as to how the results should be interpreted (is there a threshold for an exercise programme to be climate friendly?), and whether or not the different dimensions of the checklist should be weighted equally. We thus have to acknowledge that, at this stage, the checklist might rather be an inspirational tool and additional work would be needed to test an implementation of the checklist at the local level.

It is important to note that differences in local contexts might make it difficult or even impossible to implement various parts of the checklist. For example, depending on climate and cultural habits, outdoor exercise classes might be well suited in some localities or countries, while posing a health hazard or being culturally inappropriate in others. Additionally, the checklist was implicitly designed for more urban areas. To better reflect differences in context in rural areas, it may need to be adapted. For example, access to public transportation in rural areas may be limited and active transport might not be feasible due to longer distances that need to be covered.

In addition, while some dimensions of the checklist might be rather easy to sell to people participating in an exercise programme (e.g. people might be open to the message that active transport is good for the climate and their health), other dimensions might be more difficult to convey. For example, people might feel infringed upon by exercise instructors talking about sustainable clothing. This could potentially result in individuals avoiding certain exercise programmes that might implement the checklist, which would be difficult to accept from a public health perspective.

### Limitations

This study has some limitations. In applying the concept of transformative research,^16^ we have focused on the more outward-looking quality criteria of transparency and reflexivity in our methodological approach and took a normative stance to develop such a checklist. To some, the development of such a checklist might seem premature. However, with its specific focus on climate action, we feel in line with the UNs’ Sport for Climate Action Framework and call for action. While this is a highly relevant perspective, it does not reflect on the social and ecological sustainability of exercise programmes. However, additional tools exist that support the sport sector to contribute to achieving the SDGs.^50^

### Intersection between sport and sustainability

All in all, our proposed checklist is intended to stimulate discussions about the sports sector’s contribution to reducing global carbon emissions. In light of recent calls that we are facing a climate emergency,^51^ it seems certain that the sports sector, as all societal sectors, will be forced to decrease its carbon footprint substantially in the upcoming years. This will most certainly lead to difficult discussions within the sports sector. These discussions will concern mega-events of elite sports as well as recreational sports and exercise. For example, based on its high environmental burden,^52^ we anticipate discussions in the sport sector about whether alpine skiing should be practiced at all, especially in areas that rely on artificial snow. Potentially, these discussions will also spill over into other types of exercises that have a rather high carbon footprint, e.g. exercises that use heated indoor pools,^31^ but at the same time serve important health (exercise on prescription and rehabilitation programmes) and educational (swimming lessons for schoolchildren) purposes.

Ultimately, while the sport sector can make its own contributions to reduce greenhouse gas emissions, some aspects will require the support of other sectors. The transport sector is an important ally in this regard to improve the infrastructure for cycling and walking, as is architecture and urban design when it comes to the planning of sustainable sport facilities that are located near residential areas. When it comes to the re-organization of the sport sector to lower its carbon footprint, our checklist thus can be seen as a first step.

## Funding

This research was conducted as part of a project funded by the German Federal Ministry of Health (ZMVI1-2521WHO001). The ministry was neither involved in writing this manuscript nor in the decision to submit the article for publication.

## Disclaimer

The writing group takes sole responsibility for the content of this article, and the content of this article reflects the views of the authors only. K.W. and S.W. are staff members of the WHO. The authors alone are responsible for the views expressed in this publication, and they do not necessarily represent the decisions or the stated policy of the WHO.

*Conflicts of interest*: None declared.

Key points

- A 16-item checklist for climate friendly sport and exercise programmes was developed to facilitate the implementation of the United Nations Sport for Climate Action Framework at local level.
- Local level stakeholders might consider the following aspects when setting up climate-friendly sport and exercise programmes: active transport, the carbon footprint of different types of exercises, sport clothing and equipment, instructors as champions for climate action, and advertising and communication.
- The implementation of the checklist would be an important next step to identify differences in local contexts as well as facilitators and barriers.
- Furthermore, the checklist might stimulate discussions about the sports sector’s contribution to reducing global carbon emissions.
